# Safety and Efficacy of Two Trabecular Micro-Bypass Stents as the Sole Procedure in Japanese Patients with Medically Uncontrolled Primary Open-Angle Glaucoma: A Pilot Case Series

**DOI:** 10.1155/2017/9605461

**Published:** 2017-02-07

**Authors:** Daisuke Shiba, Shingo Hosoda, Saori Yaguchi, Naoki Ozeki, Kenya Yuki, Kazuo Tsubota

**Affiliations:** Department of Ophthalmology, Keio University School of Medicine, Tokyo, Japan

## Abstract

*Purpose*. To evaluate efficacy and safety of a trabecular micro-bypass stent system when used as the sole procedure in Japanese patients with medically uncontrolled primary open-angle glaucoma (POAG).* Design*. Prospective nonrandomized interventional pilot study.* Methods*. Ten eyes of 10 Japanese patients with medically uncontrolled POAG taking three ocular hypotensive medications were treated using only the implantation of two iStent trabecular micro-bypass stents. Each patient continued to take the same ocular hypotensive medications used preoperatively throughout the study. Intraocular pressure (IOP) and endothelial cell density (ECD) were determined at baseline and at 1, 3, and 6 months postoperatively. Best-corrected visual acuity (BCVA) was measured at baseline and 6 months after surgery.* Results*. Mean IOP was 22.0 ± 3.0 mmHg at baseline and 16.9 ± 3.6 mmHg at 6 months, which represented a mean reduction of 5.1 mmHg or 23.2%. No significant changes were observed in the ECD and BCVA. Complications that occurred during the early postoperative period included hyphema, peripheral anterior synechiae, and occlusion of the stent by the iris.* Conclusion*. Implantation of two trabecular micro-bypass stents as the sole procedure in Japanese POAG patients effectively reduced IOP and exhibited a favorable safety profile. Clinical Trials Registration number is UMIN000004002.

## 1. Introduction

The goal of glaucoma therapy is to halt the progression of the disease through reduction of the intraocular pressure (IOP) in the safest manner possible. In some glaucoma patients, however, combination therapy using antiglaucoma medications cannot sufficiently achieve the target pressure. In such cases, while laser trabeculoplasty is considered safe, the procedure may have a limited efficacy and durability, and it can also be associated with pain and inflammation [1]. We consider laser trabeculoplasty as an adjunctive to medical treatment, not a replacement of the incisional surgery. However, while glaucoma filtration surgery does provide a significant reduction in the IOP, this procedure is generally reserved for more serious cases due to potential risks such as endophthalmitis, suprachoroidal hemorrhage, and hypotony maculopathy [2–4].

Microinvasive glaucoma surgeries (MIGS) were developed to address the need for a less invasive surgical approach to lowering IOP. The iStent trabecular micro-bypass procedure (Glaukos Corp.) is the first of this class of new ab interno devices that may significantly reduce the IOP while maintaining a high safety profile [5]. The first generation of the iStents was the titanium, L-shaped stent (GTS100), which was designed to create a patent bypass through the trabecular meshwork in order to facilitate the natural outflow of the aqueous humor, thereby lowering the IOP [6]. Although the iStent has been investigated in a number of worldwide studies over the past several years [7–16], only a few have reported on the efficacy of a single iStent (GTS100) or two stents when used as the sole procedure [13, 16, 17]. The present study was designed to assess the IOP-lowering effect and safety when using two iStents (GTS100) as the sole procedure in medically uncontrolled primary open-angle glaucoma (POAG).

## 2. Subjects and Methods

### 2.1. Patients and Study Design

This was a prospective, noncomparative, nonrandomized, single-center consecutive case series involving 10 eyes from 10 patients with medically uncontrolled POAG. In this case series, patients were defined as being “medically uncontrolled” if they had both a progressive visual field disturbance and an intraocular pressure of 18 mmHg or higher while on a regimen of three ocular hypotensive eye drops that included a prostaglandin analogue, beta-blocker, and carbonic anhydrase inhibitor. At the time of this study, these regimens were considered to be the maximal tolerable topical glaucoma therapy in Japan. Based on our experience of conventional trabeculotomy, bibliographic information, and our unpublished work on trabecular micro-bypass stents, we believed that there was no reason to include any exclusion criteria regarding visual field loss. However, we did not include any patient that had any risk of the loss of useful central vision due to poor postoperative IOP control. In addition, individuals were excluded if they had previous argon laser trabeculoplasty, selective laser trabeculoplasty, or intraocular surgery (with the exception of cataract surgery).

This study was approved by the Keio University School of Medicine Ethics Committee and written informed consent was obtained from each patient. This clinical trial is registered with the University Hospital Medical Information Network (UMIN000004002).

Patient demographics, IOP, corneal endothelial cell density (ECD), and best-corrected visual acuity (BCVA) were recorded preoperatively. ECD was measured with noncontact specular microscopy (FA-3509, Konan Medical) and the IOP was measured with Goldmann applanation tonometry (Haag-Streit).

### 2.2. Surgical Technique

All surgical procedures were performed by DS. A 1 mm clear corneal incision was made in the temporal quadrant. The anterior chamber was then filled with viscoelastic agent (OPEGAN Hi, Santen Pharmaceutical Co., Ltd.) to improve visualization of the angle. With the operator positioned at the temporal side of the patient, an inserter with the first stent was introduced in the anterior chamber via a clear corneal incision. Angle view was obtained with a surgical gonioprism (Ocular Hill Surgical Gonioprism, Ocular Instruments). If the right eye was operated on, the leading edge was gently slid downwardly through the lower nasal region of the trabecular meshwork into Schlemm's canal. If the left eye was operated on, the leading edge was gently slid upwardly through the upper nasal region of the trabecular meshwork into Schlemm's canal. The first stent was inserted using a clockwise rotation, with the GTS100R stent used for the insertions in either the right or left eye. If the right eye was used, a second stent was inserted upwardly (counterclockwise rotation) through a second incision made at the inferior temporal cornea into the upper nasal region of the trabecular meshwork. After inserting the stent facing upwards, it was then released from the inserter followed by removal of the inserter. The second anticlockwise insertion used the GTS100L stent. The clock positions of the stents were 2 and 4 when used in the right eye and 8 and 10 when used in the left eye. The viscoelastic agent was then removed and the anterior chamber inflated with saline solution to achieve physiologic pressure.

### 2.3. Follow-Up

Postoperative care included 0.5% levofloxacin drops (Cravit, Santen Pharmaceutical Co., Ltd.) and 0.1% betamethasone sodium phosphate eye drops (Sanbetason, Santen Pharmaceutical Co., Ltd.) 3 times a day for 1 month. Patients were instructed to restart the same preoperatively used glaucoma medications 1 day postoperatively. Thus, the patient's preoperative ocular hypotensive eye drop regimen was maintained throughout the study period.

Follow-up visits occurred at 1 day, 1 week, and at 1, 2, 3, and 6 months. Examinations performed at each of these follow-up visits included slit-lamp examination, gonioscopy, and measurement of the IOP. In addition, specular microscopy was performed at 1, 3, and 6 months, while the BCVA was checked at 1 day, 1 week, and 1 and 6 months.

### 2.4. Data Analysis

Statistical analyses were carried out using the IBM SPSS Statistics 20 software. The paired *t*-test was adapted to a single comparison between the preoperative BCVA and the BCVA at 6 months. In patient exited from the study before the 6 months' visit, the last available BCVA was used by last observation carried forward method. Dunnett's procedure was used as a multiple comparison procedure for the analysis of the IOP and ECD before surgery versus at each postoperative visit. *P* value of <0.05 was considered significant.

## 3. Results

This study investigated 10 eyes (including 3 pseudophakic eyes) of 10 patients (7 male, 3 female), all of whom were Japanese. The mean age was 64.6 ± 10.7 years. Three ocular hypotensive topical medications were prescribed for each of the included eyes. While taking these medications, the mean preoperative IOP was 22.0 ± 3.0 mmHg. Mean preoperative ECD was 2506 ± 570 cells/mm^2^ and mean preoperative BCVA was −0.014 logMAR. The Shaffer grades determined for the preoperative ocular angle opening were 3 in 1 phakic eye and 4 in the other 9 eyes. The average of the mean deviation values for the Humphrey field analyzer 30-2 was −15.4 ± 8.1 dB. Three patients that exhibited severe visual field disturbance had already lost their useful central visual field. The surgery was uneventful in all eyes, with all of the stents successfully implanted upon the first or second attempt. Eight patients completed 6 months of follow-up. One pseudophakic patient was lost to follow-up at 3 months due to other health problems. As 1 pseudophakic patient underwent a trabeculectomy at 4 months after the stent implantation, our analysis included the data from before but not after the trabeculectomy. Although patient exhibited an IOP reduction for a month, his IOP at 1 month postoperatively was 18. In addition, even though the stents were implanted correctly and were not obstructed by the iris or anything else, there was reelevation of his IOP to 26 and 24 mmHg at 2 and 3 months postoperatively.

**Figure 1:**
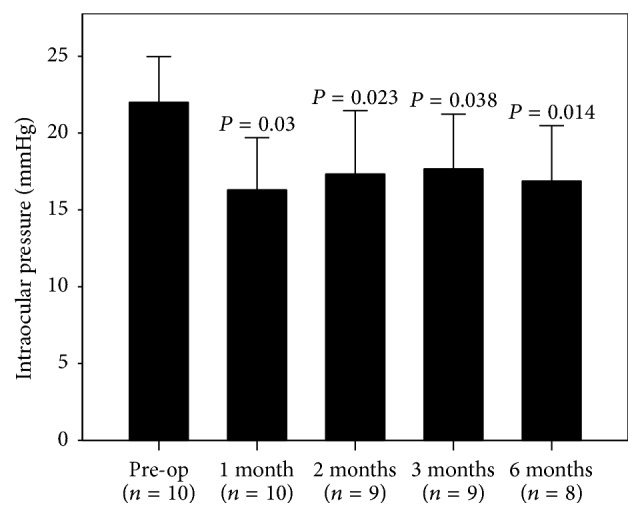
Mean intraocular pressure (+ standard deviation) at each study visit. The observed decrease in the mean intraocular pressure for all of the postoperative visits was statistically significant from the preoperative intraocular pressure values (*P* < 0.05, Dunnett's procedure versus pre-op).

**Figure 2:**
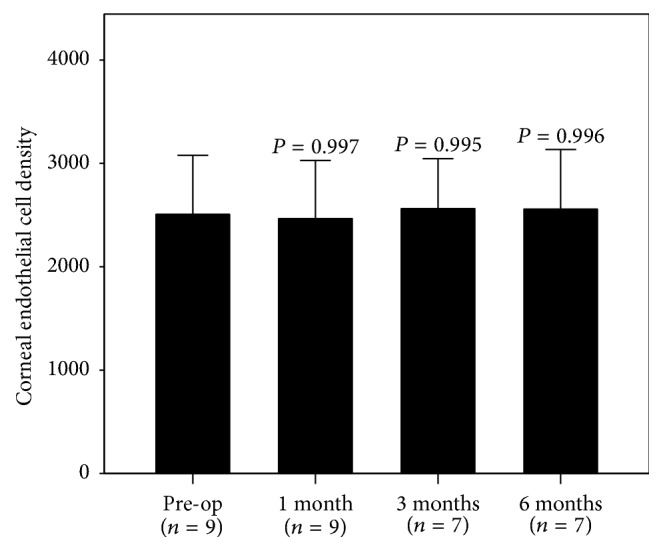
Mean corneal endothelial density (+ standard deviation) at each study visit. No statistically significant change from the preoperative corneal endothelial density was noted (Dunnett's procedure versus pre-op).

**Figure 3:**
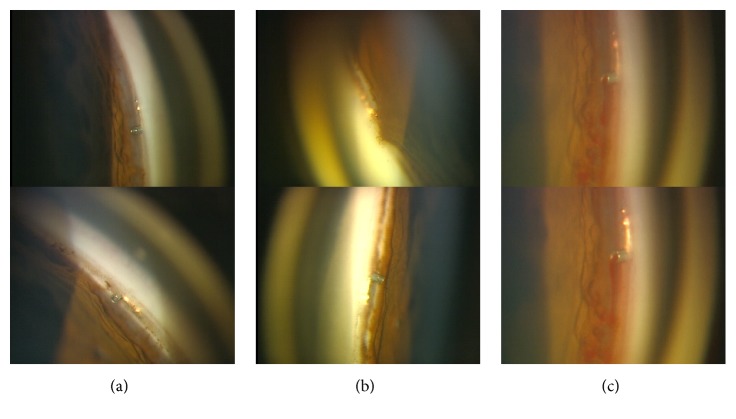
Gonioscopical photographs after two iStents' implantation in the present study. (a) The upper stent (GTS100R) in the left eye was implanted upwardly using clockwise rotation, that is, against gravity. No malposition was observed. The lower photograph of the same eye shows the lower stent (GTS100L) implanted downwardly using counterclockwise rotation. (b) Obstruction due to the iris was only observed in one of the two stents. The upper stent (GTS100L) in the right eye is obstructed, while the inlet of the lower stent (GTS100R) is open. (c) Blood reflux from the upper stent (GTS100R) was observed in the same eye as (a) immediately after the placement of the gonioscopy. The upper and lower photographs show the time course.

Mean IOP dropped to 16.1 ± 3.5 mmHg at 1 month postoperatively. The reduction in pressure was maintained at 6 months, with a mean IOP of 16.9 ± 3.6 mmHg (Figure 1, Supplementary Table 1 in Supplementary Material available online at https://doi.org/10.1155/2017/9605461). This represents a clinically and statistically significant reduction in the IOP of 5.1 mmHg from the preoperative mean IOP (*P* < 0.05). At 6 months, the IOP reduction rate was 23.2%. No significant changes were observed for either the ECD (Figure 2) or the BCVA. Mean ECD at 6 months was 2556 ± 577 cells/mm^2^ compared to 2506 ± 570 cells/mm^2^ at baseline. At last observation, the mean BCVA was −0.0059 ± 0.11 logMAR, compared to −0.0014 ± 0.11 logMAR at baseline (*n* = 10, *P* = 0.34). No stent malposition was observed throughout the study period (Figure 3(a)).

Postoperative complications included microhyphema (only observed by gonioscopy) in 3 eyes and mild hyphema in 1 eye, all of which resolved without intervention by the 1-month visit; transient IOP elevation of 32 mmHg on the next day following the surgery in 1 eye, which resolved spontaneously; peripheral anterior synechiae (PAS) in 4 eyes, which was treated with argon laser gonioplasty (LGP); and occlusion by the iris, which occurred in 2 eyes of the LGP-treated eyes with PAS by the 1-month visit and in 1 eye of the LGP-treated eyes with PAS at the 2-month visit (Table 1, Figure 3(b)). These occlusions were observed only in phakic eyes. Blood reflux from the stent was often observed in a few eyes for a week postoperatively (Figure 3(c)). There was no evidence of hypotony, flat anterior chamber, choroidal detachment, or endophthalmitis in any of the eyes.

## 4. Discussion and Conclusion

Starting in 1960, attempts have been made to reduce the resistance of the trabecular outflow pathway. Trabeculotomy ab externo was one of such attempts and is one of the more representative procedures [18, 19]. The IOP-lowering effect of trabeculotomy in adult patients was first reported in a study by Tanihara et al. [20]. Recently, Chin et al. reported finding a much better IOP-lowering effect of modified 360-degree suture trabeculotomy using 5-0 nylon suture [21]. Even though trabeculotomy has been considered to be a safe and effective surgical method, its invasiveness to the conjunctiva and sclera has prevented the method from becoming globally widespread. The trabecular micro-bypass stent system theoretically provides the same IOP-lowering mechanism as trabeculotomy, with the advantage of having a much smaller invasiveness than that for trabeculotomy. Out of the current MIGS, we found that implantation of two trabecular micro-bypass stents clinically and significantly reduced the IOP even when used as the sole procedure over 6 months postoperatively.

Several previous studies have also published surgical results for the trabecular micro-bypass procedure. However, since most of these compared trabecular micro-bypass surgery combined with cataract extraction to cataract extraction solo surgery, this made it difficult to determine the outcomes and the genuine effect of the trabecular micro-bypass because of the variable ocular hypotensive medications and the IOP reduction associated with cataract extraction itself. Samuelson et al. reported on the use of single trabecular micro-bypass stent surgery combined with cataract surgery [8]. In their report, single trabecular micro-bypass stent implantation induced a 20% increase in patients free of medication as compared to the cataract surgery group. Also, patients in the iStent group reported a mean reduction of 1.4 hypotensive medications versus 1.0 medication in the cataract surgery only group, representing a difference in medication reduction of 0.4 medications. On the other hand, Fernández-Barrientos et al. utilized the two trabecular micro-bypass stents' surgery approach combined with cataract surgery [9]. Their findings showed that the implantation of two iStents resulted in an additional 4.0-mmHg reduction in the IOP and an additional 0.4 decrease in the glaucoma eye drops compared with the cataract surgery group at 6 months. Comparable to their results, our present study that used two stents also showed a 5.1-mmHg decline in the IOP without any medication change.

Prospective studies on the use of the second generation of iStent (iStent inject: GTS400) as a solo procedure have been reported by two researchers [22, 23]. Although the GTS400 was designed to enhance trabecular outflow in the same way as for the GTS100, it has been modified in order to make it easier to implant and achieve an enhanced IOP reduction when performing a surgery with two stents. Since one GTS400 applicator contains two stents, we expect the results for the GTS400 will be similar to that found for the present study that also implanted two stents. These previous studies also reported washout IOP decreases of 13.0 mmHg and 10.6 mmHg from the baseline washout IOPs of 25.2 mmHg [22] and 26.3 mmHg [23], respectively. A retrospective study of the iStent inject (GTS400) when used as a solo procedure additionally reported similar outcomes in POAG or pseudoexfoliation glaucoma eyes [24]. The different design of the iStent inject (GTS400) from the iStent (GTS100) might result in a different outcome profile from iStent. Further studies that address the generation difference of iStent will need to be undertaken. More recently, a comparative study of one, two, or three trabecular micro-bypass stents (GTS100) in POAG eyes was published by Katz et al. [17]. In this report, when two-stent implantation surgery was used as a solo procedure, the mean IOP decreased by 6.6 mmHg from the preoperative 20.1 mmHg on 1.76 medications at 6 months. Out of 41 eyes in the two-stent group, only 4 eyes required the addition of medications postoperatively. Compared to these previous studies, the 5.1 mmHg IOP reduction noted in our current study when using the same medications seems to be slightly smaller. We speculate that there are two reasons for this discrepancy. First, as our patients had a mean preoperative IOP of 22.0 mmHg under three medications, they should have had a much higher baseline washout IOP and more severe dysfunction in the aqueous outflow pathway. Second, our study included 3 phakic cases that exhibited stent occlusion due to the iris in one of the two stents. Due to this occlusion, the enhancement of the aqueous outflow was thought to have deteriorated to some extent. Thus, if we only take the 7 phakic eyes into consideration, this results in nearly half of the eyes implanted with two stents undergoing occlusion due to the iris. The reason for this is most likely related to having performed the surgery in phakic eyes of Asian patients. Buchacra et al. reported finding no incident of stent occlusion due to the iris in 10 eyes, which included 7 phakic eyes [16]. However, there was no description of the race of the patients included in their study. Moreover, in a study of Caucasian phakic eyes in a clinical setting that was similar to ours, there was no mention of any occlusion due to the iris [17]. Far East Asians have been reported to have a narrower ocular angle in general [25]. Thus, it is rational to expect that there would be more occlusions due to the iris in Japanese eyes versus Caucasian eyes. Although the prophylactic use of LGPs to target the adhesions associated with PAS was performed in order to prevent occlusion of the stents after PAS had arisen, these treatments could not effectively preclude the occlusions of the stent, thereby resulting in PAS enlargement. The management of PAS or stent occlusion will need to be examined in a future study.

Originally, the trabecular micro-bypass stent system was designed to be used in combined surgery with cataract extraction. Nonetheless, some surgeons showed an interest in the solo use of the system, and some studies, including the present study, have reported finding significant IOP reductions. Although our study only included a small group of patients, our findings indicate that care should be taken when performing trabecular micro-bypass stenting surgery in the phakic eye, especially in patients of the Asian race.

Based on the results of our current study, we speculate that the implantation of two stents was able to relieve the unfavorable effect of the obstruction by the iris. A multiple stents surgery can reduce the probability of obstructions in all of the stents. Consequently, surgeries using multiple stents or combined surgeries with cataract extraction might be advisable in cases with shallow angled eye.

Since any artificial object in the anterior chamber could potentially cause damage to the corneal endothelium such as the anterior chamber intraocular lens, confirmation on whether trabecular micro-bypass stents impair the corneal endothelium needs to be determined [26]. At the present time, there has been no published data on the safety of using trabecular micro-bypass stenting with regard to the corneal endothelium. However, when the Hydrus stent procedure was combined with cataract extraction, Fea et al. reported finding no additional damage to the corneal endothelium as compared to cataract alone [27]. In the present study, the mean ECD was maintained throughout the study. While the number of cases in the present study was too small to demonstrate our proposed thesis, our results do reinforce the safety of the corneal endothelium after the trabecular micro-bypass stent implantation procedure.

There were some limitations for the current study, especially regarding the small number of patients and the short duration of the follow-up due to the fact that this was a pilot study. Thus, due to the small patient number and short study duration, we cannot definitively determine whether trabecular micro-bypass stenting solo surgery should be adopted for routine glaucoma management based on the current results. However, there have been a few previous studies that have reported finding a long-term stability for the IOP reduction when using the trabecular micro-bypass stent [12, 14]. Based on the results of these prior studies in conjunction with our pilot study, a future study with a larger number of patients and longer follow-up will need to be undertaken in order to confirm our present results.

The current study is the first assessment of the iStent (GTS100) when used as a sole procedure in Asian POAG eyes. The current efficacy and safety results are moderately consistent with several earlier published studies on solo surgery or combined surgery with cataract extraction. Based on these findings, we conclude that the iStent (GTS100) is effective in providing significant IOP reduction while maintaining a favorable safety profile, even if used as the sole procedure.

## Supplementary Material

Intraocular pressure (mmHg) in each patient at each study visit is shown.

## Figures and Tables

**Table 1:** Postoperative complications.

Complication	*N*
Hyphema	4
Microhyphema^*∗*^	3
Hyphema	1
IOP ≥ 30 mmHg	1
Peripheral anterior synechiae	4
Occlusion by iris	3

^*∗*^Microhyphema only observed by gonioscopy.
