# Supplementary material for: Safety and Efficacy of Two Trabecular Micro-Bypass Stents as the Sole Procedure in Japanese Patients with Medically Uncontrolled Primary Open-Angle Glaucoma: A Pilot Case Series
Source: J Ophthalmol. 2017 Feb 7;2017:9605461. doi: 10.1155/2017/9605461 (PMC5318622; doi:10.1155/2017/9605461)
Supplement: Supplementary file 1 — Intraocular pressure (mmHg) in each patient at each study visit is shown. [file 9605461.f1.docx]

**Supplementary Table 1: Intraocular Pressure (mmHg) in Each Patient**

| Patient | Pre-op | 1 Month | 2 Months | 3 Months | 6 Months |
| --- | --- | --- | --- | --- | --- |
| 1 | 28 | 21 | 20 | 21 | 20 |
| 2* | 24 | 18 | 26 | 24 |  |
| 3 | 21 | 14 | 14 | 16 | 19 |
| 4 | 19 | 17 | 16 | 16 | 15 |
| 5 | 22 | 18 | 19 | 19 | 19 |
| 6 | 22 | 19 | 19 | 20 | 22 |
| 7 | 18 | 10 | 15 | 16 | 15 |
| 8† | 23 | 13 |  |  |  |
| 9 | 19 | 14 | 13 | 13 | 12 |
| 10 | 24 | 19 | 14 | 14 | 13 |

*Trabeculectomy was performed at postoperative 4 months.

†Loss of follow up after postoperative 1 month.
